# Maintenance of Mitochondrial Morphology in Cryptococcus neoformans Is Critical for Stress Resistance and Virulence

**DOI:** 10.1128/mBio.01375-18

**Published:** 2018-11-06

**Authors:** Andrew L. Chang, Tamara L. Doering

**Affiliations:** a Department of Molecular Microbiology, Washington University School of Medicine, Washington University, St. Louis, Missouri, USA; Duke University Medical Center

**Keywords:** Cryptococcus neoformans, Mitochondria, Mitochondrial morphology, Pathogenic fungi

## Abstract

Mitochondria are essential organelles that act in pathways including ATP production, β-oxidation, and clearance of reactive oxygen species. They occur as a complex reticular network that constantly undergoes fusion and fission, mediated by dynamin-related proteins (DRPs). DRPs include Fzo1, which mediates fusion, and Dnm1, Mdv1, and Fis1, which mediate fission. Mitochondrial morphology has been implicated in virulence in multiple fungi, as with the association between virulence and increased mitochondrial fusion in Cryptococcus gattii. This relationship, however, has not been studied in Cryptococcus neoformans, a related opportunistic pathogen. C. neoformans is an environmental yeast that can adapt to the human host environment, overcome the innate immune system, and eventually disseminate and cause lethal meningoencephalitis. We used gene deletion of key DRPs to study their role in mitochondrial morphology and pathogenesis of this yeast. Interestingly, increasing mitochondrial fusion did not increase resistance to oxidative stress, unlike in model yeast. Blocking mitochondrial fusion, however, yielded increased susceptibility to oxidative and nitrosative stresses as well as complete avirulence. This lack of virulence was not mediated by any effects of altered mitochondrial function on two major virulence factors, capsule and melanin. Instead, it was due to decreased survival within macrophages, which in turn was a consequence of increased susceptibility to oxidative and nitrosative stress. Supporting this conclusion, reactive oxygen species (ROS) scavengers rescued the ability of fusion mutants to survive intracellularly. These findings increase our understanding of cryptococcal biology and virulence and shed light on an important group of proteins and cellular processes in this pathogen.

## Abstract (precis)

C. neoformans is a yeast that causes fatal brain infection in close to 200,000 people worldwide every year, mainly afflicting individuals with AIDS or others who are severely immunocompromised. One feature of this microbe that helps it cause disease is that it is able to withstand toxic molecules it encounters when host cells engulf it in their efforts to control the infection. Mitochondria are important organelles responsible for energy production and other key cellular processes. They typically exist in a complex network that changes morphology by fusing and dividing; these alterations also influence mitochondrial function. Using genetic approaches, we found that changes in mitochondrial morphology dramatically influence cryptococcal virulence. We showed that this occurs because the altered mitochondria are less able to eliminate the harmful molecules that host cells produce to kill invading microbes. These findings are important because they elucidate fundamental biology and virulence and may suggest avenues for therapy.

## INTRODUCTION

Mitochondria likely originated 2 billion years ago when an alphaproteobacterium was engulfed by another single-celled organism, creating the first prototype of a eukaryotic cell (1, 2). Divergent evolution since then has generated notable differences in mitochondria across eukaryotic lineages, such as variation in the distribution of genes encoding mitochondrial proteins between these organelles and the nucleus and in mechanisms of mitochondrial DNA (mtDNA) structure and coding (3–5). However, all mitochondria share critical functions, including ATP production, β-oxidation, apoptosis, and oxidative stress clearance (3).

Mitochondria are dynamic organelles that interact to create a cellular network that undergoes constant remodeling, adopting multiple morphologies (6, 7). During healthy growth, mitochondria exhibit a diffuse morphology, forming a reticulum distributed throughout the cell (8). Under stress, however, mitochondria may fuse to form what is termed tubular morphology, which increases the ability of cells to resist stress that might otherwise be lethal. Fusion may allow mitochondria to share mtDNA and thus survive the mtDNA damage caused by oxidative stress (9), and to share components of the mitochondrial proteome (10). While increased mitochondrial fusion may be temporarily beneficial, unopposed fusion can also result in dysregulated mitochondrial and mtDNA inheritance (6). Inhibited fusion or unopposed fission yields another morphology, fragmentation of the mitochondrial network (11). This leads to cellular damage, loss of the mitochondrial membrane potential, and eventually apoptosis mediated by cytochrome *c* release. The morphology of the mitochondrial network thus hangs in a delicate balance that is influenced by cell context.

In Saccharomyces cerevisiae, fusion of the mitochondrial network is mediated by two dynamin-related proteins (DRPs), Fzo1 and Mgm1. Although little is known about exactly how these “mitofusins” function, it is believed that the fusion machinery creates contact sites between mitochondria (12, 13). Interestingly, other DRPs mediate fission of the mitochondrial network; in S. cerevisiae, these include Fis1, Mdv1, and Dnm1 (1). During fission, Fis1 on the surface of mitochondria is bound by Mdv1. Mdv1 then promotes the assembly of Dnm1 into a ring-like structure, permitting scission of membranes and fission of the organelle (14, 15).

Mitochondrial morphology has been implicated in virulence-related traits of multiple fungal pathogens (16). For example, in Candida albicans, deletion of the mitofusin Fzo1 results in a decreased ability to adopt a tubular morphology and increased susceptibility to peroxide stress and azole drugs (17). In Aspergillus fumigatus, the deletion of genes encoding fission DRPs causes decreased growth rates compared to those of wild-type (WT) cells (18). Mitochondrial morphology has also been implicated in fungal pathogenesis in Cryptococcus gattii, where the ability to tubularize mitochondria correlates with virulence in humans (19, 20).

Our focus is the opportunistic yeast Cryptococcus neoformans, a pathogen that is ubiquitous in the environment. Cryptococci are acquired by inhalation and first interact with the innate immune system in the lung, where they adapt to the host environment and may remain in a latent state. If the infected individual is or becomes immunocompromised, however, the cryptococcal cells may disseminate throughout the body, showing a tropism for the brain (21). The resulting lethal meningoencephalitis annually kills approximately 200,000 people worldwide, with extremely high mortality in sub-Saharan Africa (22, 23).

Mitochondrial function is important for cryptococcal virulence (24, 25), but we wondered whether and how mitochondrial morphology influences cryptococcal pathogenesis. To probe this relationship between form and function, we deleted genes that encode the major actors in the normal maintenance of mitochondrial morphology. We found that altering mitochondrial morphology in this way affected the susceptibility of C. neoformans *in vitro* to multiple stresses that are encountered during infection. Consistent with this behavior, mitochondrial morphology mutants were also impaired in their ability to cause disease *in vivo*. Finally, we showed that the reduction in virulence is primarily mediated by defects in the clearance of reactive oxygen species.

## RESULTS

**FIG 1:**
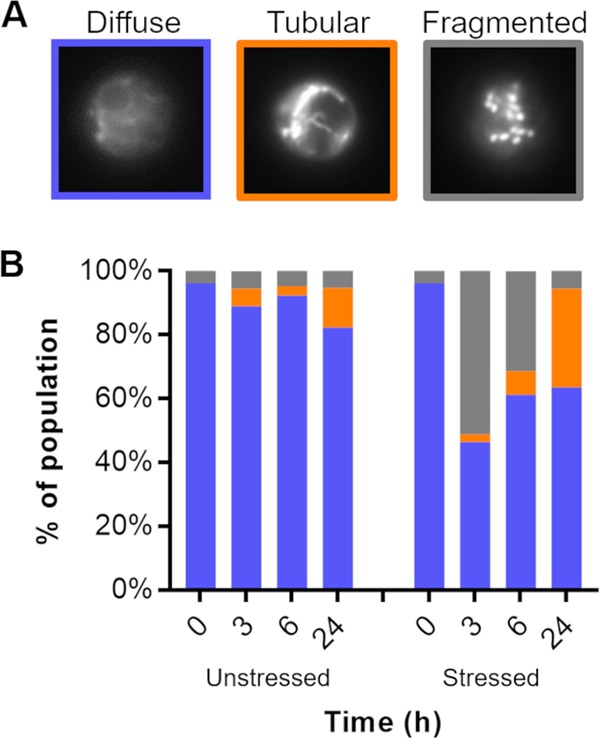
*C. neoformans* mitochondrial morphology without and with oxidative stress. (A) Examples of the indicated mitochondrial morphologies, imaged by fluorescence microscopy of cells stained with MitoTracker. (B) Distribution of mitochondrial morphology over time in cells grown in DMEM (37°C, 5% CO_2_) without (left) or with (right) 1 mM H_2_O_2_ as a stressor. At each time point, >150 cells were categorized per sample and categorized in a double-blinded manner.

To assess the relationship between mitochondrial morphology and cryptococcal pathogenesis, we first needed to characterize mitochondria in C. neoformans. To do this, we labeled wild-type (WT) cryptococcal cells with a mitochondrion-specific fluorescent dye, MitoTracker CMXRos. We observed that cryptococcal mitochondria adopt diffuse, tubular, and fragmented morphologies (Fig. 1A), similar to mitochondria in model organisms.

We next assessed the distribution of morphology across populations of cryptococcal cells, using a blinded experimental design to avoid any unconscious bias in scoring. We found that by 1 day after transfer from rich yeast medium (yeast extract-peptone-dextrose [YPD]) at 30°C to the mild stress of tissue culture growth conditions (Dulbecco’s modified Eagle medium [DMEM] at 37°C, 5% CO_2_), a small fraction of C. neoformans cells (12%) had transitioned their mitochondrial network toward the tubular morphology (Fig. 1B, left), while only 5% were fragmented. The addition of oxidative stress (1 mM H_2_O_2_) to these growth conditions had a much more dramatic effect. By the end of 3 h, 50% of the cells had entered the fragmented mitochondrial state, with only 2% being tubular. With time, the population recovered, however, with a steady reduction in the fraction of cells with fragmented morphology and an increase in cells with tubular morphology (Fig. 1B, right).

Having established the overall pattern of mitochondrial morphology in C. neoformans, we next attempted to alter this balance by genetic manipulation, starting with perturbation of fusion. We first used BLASTp to search the C. neoformans genome for predicted proteins homologous to S. cerevisiae Fzo1, a key mitofusin. We found a predicted ortholog (CNAG_06688), which we termed Fzo1. Fzo1 in C. neoformans and S. cerevisiae share 32% amino acid identity, with an E value of 2.63 × 10^−63^.

**FIG 2:**
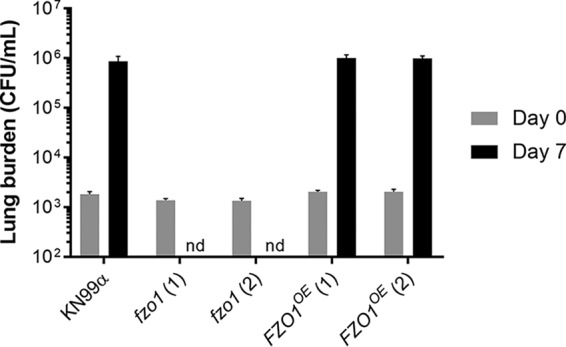
Mitochondrial fusion mutants do not survive in mice. Shown is mouse lung burden after intranasal infection (see Materials and Methods) with the indicated fungal strains; two independent isolates are shown for the *fzo1* and *FZO1^OE^* mutants. Gray, day 0 (*n* = 2); black, day 7 (*n* = 4). Mean and standard error of the mean (SEM) are shown. nd, not detected.

To examine the role of cryptococcal Fzo1, we both deleted and overexpressed the corresponding gene in the KN99α background of C. neoformans, generating *fzo1* and *FZO1^OE^* mutant strains, respectively. As mentioned above, mitochondrial morphology has been implicated in in the virulence of the related species C. gattii, although it has been suggested that it may play a smaller role in C. neoformans (19, 20). For this reason, we first tested whether dysregulation of *FZO1* would affect C. neoformans infection, using lung burden after intranasal inoculation as a measure of virulence. We found that while lung burden increases almost three orders of magnitude within a week of infection with KN99α cells, two independent *fzo1* mutant strains were completely cleared in this interval (Fig. 2). Overexpression of *FZO1*, however, did not increase lung burden (Fig. 2).

**FIG 3:**
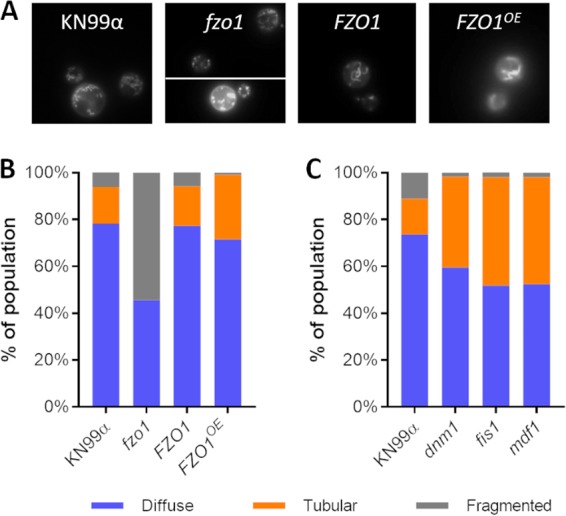
Mutant mitochondrial morphology. (A) Sample images of the strains indicated, stained as in Fig. 1. (B and C) Distribution of mitochondrial morphology, assayed as in Fig. 1, in strains engineered to alter fusion (B) or fission (C). Plots are representative of at least three independent experiments.

For further studies of cells with altered fusion capability, we generated a complemented strain (*FZO1*) to expand our strain set and examined mitochondrial morphology, as described above (Fig. 3A). We found that 54% of the deletion strain population showed the fragmented morphology, in contrast to the WT and complemented strains (6%; Fig. 3B). Interestingly, the *FZO1^OE^* mutant strain showed a 76% increase in tubular morphology cells, mainly at the expense of the fragmented cells; the diffuse population was similar to that of WT and complemented strains (Fig. 3B).

**FIG 4:**
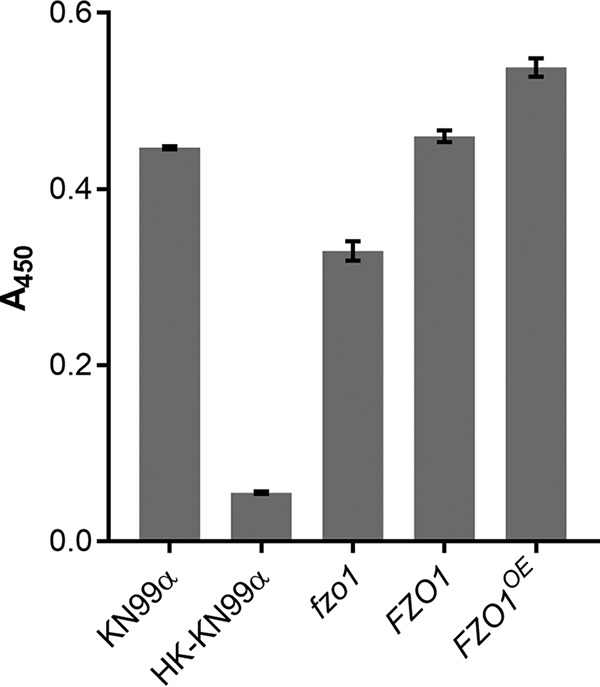
Metabolic activity of fungi. Plotted are the mean and SD of XTT assay results (*A*_450_) for the indicated strains; HK, heat-killed cells (see Materials and Methods). Results shown are representative of three biological replicate experiments.

To broadly assess mitochondrial function, we next measured succinate dehydrogenase activity in our strain set by assaying the reduction of 2,3-bis(2-methoxy-4-nitro-5-sulfophenyl)-2H-tetrazolium-5-carboxanilide (XTT) to formazan. This mitochondrial enzyme complex is a key part of both the electron transport chain and the citric acid cycle, and its activity is a general indicator of mitochondrial health (26, 27). We found that the *fzo1* mutant strain had 30% lower succinate dehydrogenase activity than the WT and complement strains (Fig. 4). Interestingly, the *FZO1^OE^* mutant strain was slightly improved (+20%; *P <* 0.01) in its ability to perform this reaction compared to WT cells, even though other fission mutants (described below), which show an even greater shift toward tubular morphology, showed no increase in metabolic activity (see Fig. S1 in the supplemental material). These results suggest that the manipulation of *FZO1* has broader effects on mitochondrial biology than simply altered morphology.

10.1128/mBio.01375-18.1 FIG S1 Metabolic activity of fungi. Plotted are the mean and SD of XTT assay results (*A*_450_) for each indicated strain; HK, heat-killed cells. Results are shown for two independently derived stains for each fission mutant, and all results are representative of three biological replicate experiments. Download FIG S1, PDF file, 0.1 MB. Copyright © 2018 Chang and Doering. 2018 Chang and Doering This content is distributed under the terms of the Creative Commons Attribution 4.0 International license.

**FIG 5:**
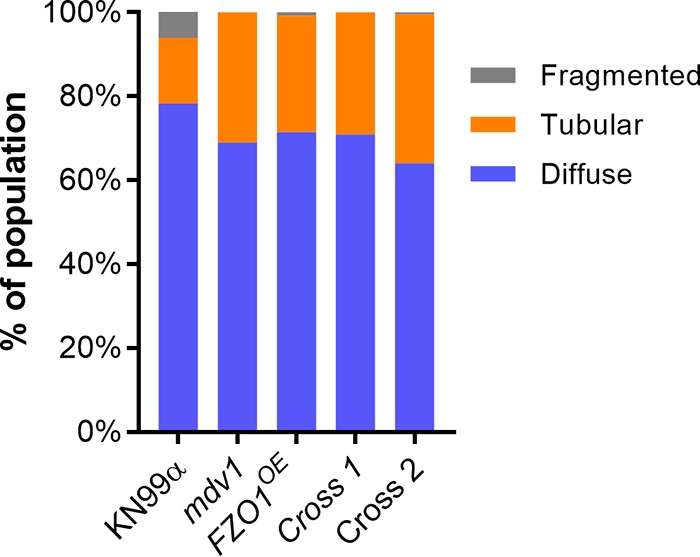
Distribution of mitochondrial morphology in strains engineered to increase mitochondrial fusion. Assays were performed as in Fig. 1, and the plot is representative of three independent experiments. Cross 1 and 2 are independent *FZO1^OE^ mdv1* double mutants.

To examine cells perturbed in the machinery for mitochondrial fission, we next generated two independent single-deletion strains for each of three DRP fission genes whose products act sequentially, *MDV1*, *DNM1*, and *FIS1*. As expected, the mutant cells were defective in fission, manifested as extremely high levels of tubular morphology relative to the WT (Fig. 3C), a more dramatic result than when we had overexpressed the *FZO1* fusion gene. Since both of these strains increase tubularization, but via independent mechanisms, we wondered whether combining their defects might further tip the balance of mitochondrial morphologies toward fusion. To test this, we performed a genetic cross of the *FZO1^OE^* mutant strain (generated in KN99α) with an *mdv1* mutant strain (generated in KN99a). (We selected this strategy over a double-deletion approach in order to perturb the mitochondrial morphology balance from both the fusion and fission pathways, rather than interfere with two DRPs involved in the same fission pathway.) We found that tubularization in the resulting double-mutant progeny was statistically similar to that of the parents (Fig. 5), suggesting a limit to this process (see Discussion).

Finally, to test whether strains defective in mitochondrial fission would show any changes in *in vivo* virulence, we used a short-term lung burden model of infection in order to assess the virulence of two independently generated deletion strains of each of the three mitochondrial fission genes. We observed no statistically significant difference from the WT in lung burden upon infection with any of the fission gene deletions. We thus focused our attention on the avirulent mitochondrial fusion mutant (Fig. S2).

10.1128/mBio.01375-18.2 FIG S2 Mitochondrial fission mutants have no defect in virulence. Mouse lung burden in CFU after intranasal infection (see Materials and Methods) with the indicated fungal strains; two independent isolates of *mdv1*, *dnm1*, and *fis1* are shown. Gray, day 0 (*n* = 2); black, day 7 (*n* = 4). Mean and SEM are shown. Download FIG S2, PDF file, 0.1 MB. Copyright © 2018 Chang and Doering. 2018 Chang and Doering This content is distributed under the terms of the Creative Commons Attribution 4.0 International license.

We first investigated potential reasons for the previously observed drastic reduction in virulence of the *fzo1* mutant strain. We first considered the possibility that this mutant had been cleared due to an inability to grow at host temperatures. However, *fzo1* mutant cells showed only subtle impairments in growth compared to the WT (manifested in both YPD at 30°C and DMEM at 37°C; Fig. S3), so this is unlikely to explain their complete inability to survive within the mouse lung. Increased tubularization in the context of fission mutants also did not improve growth relative to the WT (Fig. S3), suggesting that mitochondrial morphology does not significantly predict growth rate.

10.1128/mBio.01375-18.3 FIG S3 Growth curves in the indicated media of strains with altered *FZO1* expression (A) or deletions of genes involved in mitochondrial fission (B). YPD, YPD medium at 30°C, room air; RPMI, RPMI medium at 37°C, 5% CO_2_. The mean and SD of triplicate time points are shown. Download FIG S3, PDF file, 0.2 MB. Copyright © 2018 Chang and Doering. 2018 Chang and Doering This content is distributed under the terms of the Creative Commons Attribution 4.0 International license.

**FIG 6:**
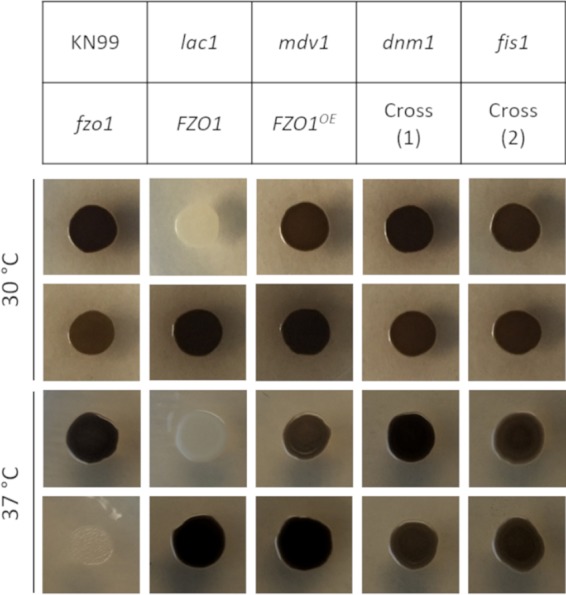
Melanization of mitochondrial morphology mutants. The indicated strains (each 1E4 cells) were grown at 30 and 37°C on l-DOPA agar to assess melanin production (see Materials and Methods for details). The *lac1* control strain cannot melanize under any conditions. Cross 1 and 2 are independent *FZO1^OE^ mdv1* double mutants.

We considered that alterations in mitochondrial morphology, and thus, function, might indirectly influence cryptococcal virulence factors. The best known of these is a large polysaccharide capsule, which both modulates the host immune system and presents a physical barrier to uptake by phagocytes. However, the capsules produced by strains altered in fission and fusion (*fzo1*, *FZO1*, *FZO1^OE^*, *mdv1*, *dnm1*, *fis1*, and *FZO1^OE^ mdv1* double mutants) were all comparable to those of WT cells (Fig. S4). Another well-known cryptococcal virulence factor is the ability to produce melanin, a dark pigment that increases resistance to reactive oxygen species and other environmental insults (28, 29). The *fzo1* mutant strain showed a marked inability to produce melanin at higher temperatures, which could increase its susceptibility to stress under these conditions (Fig. 6) (28, 30).

10.1128/mBio.01375-18.4 FIG S4 India ink stain of cells grown under capsule-inducing conditions (DMEM, 37°C, 5% CO_2_) for 24 h. Cross indicates *FZO1^*OE*^ mdv1* double mutants. Scale bar = 20 µm. Download FIG S4, PDF file, 0.9 MB. Copyright © 2018 Chang and Doering. 2018 Chang and Doering This content is distributed under the terms of the Creative Commons Attribution 4.0 International license.

**FIG 7:**
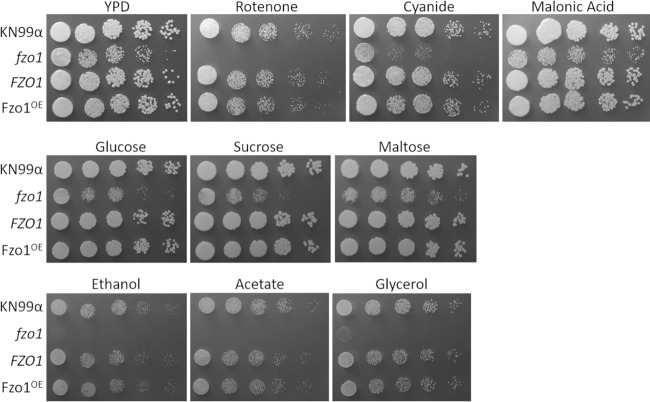
Stress phenotypes of mitochondrial morphology mutants on rich medium (YPD). Serial dilutions of the indicated strains were grown in the absence or presence of the indicated additives (see Materials and Methods for details).

**FIG 8:**
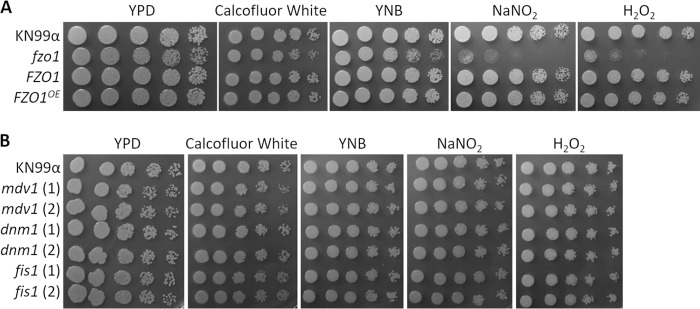
Stress phenotypes of mitochondrial morphology mutants. Serial dilutions of the indicated strains were grown in the absence or presence of the indicated stressors (see Materials and Methods for details). (A) Strains engineered to alter fusion. (B) Strains engineered to alter fission.

We next tested our mutant strains for phenotypes related to mitochondrial function, including plating on compounds that inhibit electron transport chain functions and a variety of carbon sources. We observed significant defects in the growth of the *fzo1* mutant strain on YPD supplemented with inhibitors of the mitochondrial electron transport chain complexes I and IV, such as rotenone and cyanide (Fig. 7, top row). Compared to its growth on glucose, sucrose, and maltose (Fig. 7, middle row), this mutant was also severely defective in growth on small carbon sources, such as glycerol, ethanol, and acetate (Fig. 7, bottom row). Interestingly, we observed no similar defects in fission mutant strains compared to wild-type cells (Fig. S5), consistent with their normal function in the XTT assay (Fig. S1). Finally, we examined the susceptibilities of the mutant strains to external stresses by spotting them on compounds that induced cell wall, nitrosative, and oxidative stress (Fig. 8). Although we saw no changes in sensitivity to cell wall stress, we observed that the *fzo1* deletion strain, which is defective in tubularization, showed significantly less growth (∼10-fold) under conditions of nitrosative and oxidative stress. Normal growth was restored in the *FZO1* complemented strain (Fig. 8A), but overexpression of this gene (*FZO1^OE^*) did not increase resistance to these stressors. This suggests that although decreased tubularization (compared to WT cells) reduces cell resistance to stress and impairs mitochondrial function, increased tubularization does not enhance these properties. This conclusion is supported by the phenotypes of the fission-defective strains (the *dnm1*, *mdv1*, and *fis1* mutants), which similarly demonstrate increased tubularization (Fig. 3C) yet show WT growth in the presence of oxidative stress (Fig. 8B) and electron transport chain inhibitors (Fig. S5).

10.1128/mBio.01375-18.5 FIG S5 Stress phenotypes of mitochondrial morphology mutants on rich medium (YPD). Serial dilutions of the indicated strains were grown in the absence or presence of the indicated additives (see Materials and Methods for details). Download FIG S5, PDF file, 1.1 MB. Copyright © 2018 Chang and Doering. 2018 Chang and Doering This content is distributed under the terms of the Creative Commons Attribution 4.0 International license.

**FIG 9:**
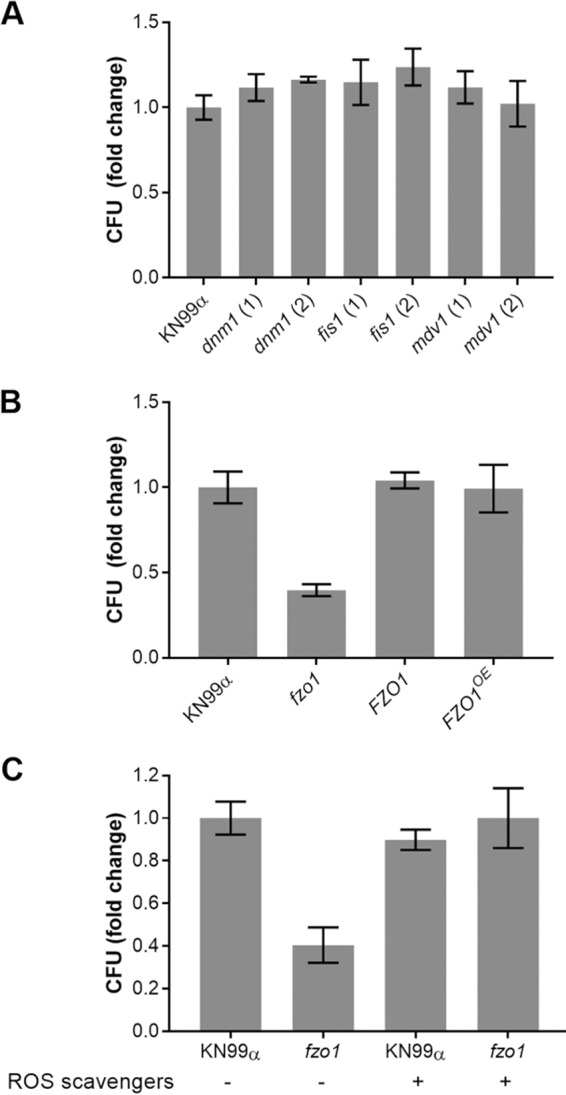
Survival of fungi after engulfment by macrophages. (A) Mutants with impaired fission. (B) Mutants with altered fusion. (C) Mutants with altered fusion in the absence and presence of ROS scavengers (see Materials and Methods). For each strain, the ratio of CFU at 48 h to CFU at 0 h was normalized to this ratio for KN99 control cells (typically 1.5 to 2.0). Mean and SD of this value are plotted, and results shown are representative of at least two biological replicate experiments.

C. neoformans is a facultative intracellular pathogen which is exposed to oxidative and nitrosative stress within host cells. We postulated that mutants that are more sensitive to such stresses *in vitro* would be compromised in their ability to survive in this environment. To test this, we allowed cryptococcal cells to be engulfed by an immortalized macrophage cell line (THP-1) and washed away free or externally adherent cells. We then assessed viable fungi by lysing the THP-1 cells and plating for CFU after varying the times of coculture. In this assay, the *dnm1*, *fis1,* and *mdv1* fission mutants, which all show increased tubularization, showed no statistical difference from WT cells (*P ≥* 0.05), though there was a slight trend toward increased survival (Fig. 9A). In contrast, the *fzo1* mutant strain, which is defective in tubularization, was markedly impaired in intracellular survival (Fig. 9B). Intracellular survival of the *FZO1* complement strain, the *FZO1^OE^* mutant, and the *FZO1^OE^ mdv1* mutant strain was like that of WT (Fig. 9B and S6). The ability of cryptococcal cells to undergo mitochondrial fusion is thus essential for their survival within host cells.

10.1128/mBio.01375-18.6 FIG S6 Survival of the indicated mutant strains that show increased tubularization after engulfment by macrophages. Crosses 1 and 2 are independent *FZO1^*OE*^ mdv1* double mutants. For each strain, the ratio of CFU at 48 h to CFU at 0 h was normalized to this ratio for KN99 control cells (typically 1.5 to 2.0). The mean and SD are plotted, and the results shown are representative of three biological replicate experiments. Download FIG S6, PDF file, 0.1 MB. Copyright © 2018 Chang and Doering. 2018 Chang and Doering This content is distributed under the terms of the Creative Commons Attribution 4.0 International license.

The intracellular survival results, coupled with our earlier plating data, suggested that the *fzo1* mutant is unable to clear reactive oxygen species (ROS) efficiently, which would also explain its defective virulence. Mannitol, superoxide dismutase, and catalase have been used experimentally to scavenge hydroxide, superoxide, and peroxide chemical species, respectively (31). We tested the effect of these ROS scavengers on cryptococcal susceptibility to intracellular killing. While the application of ROS scavengers did not increase the intracellular survival of WT cells past baseline, it did restore that of *fzo1* to WT levels (Fig. 9C).

## DISCUSSION

Mitochondrial morphology has been shown to affect the virulence of fungal pathogens, including C. albicans and A. fumigatus. Here, we have directly examined the dynamin-related proteins (DRPs) that mediate this characteristic in C. neoformans. We first used molecular manipulation to tip the balance of mitochondrial morphology toward fission. The deletion of *FZO1*, a mitofusin, yielded an expanded population of cells in the fragmented mitochondrial state which showed impaired stress resistance, similar to observations in model yeast (32). These cells also showed a striking reduction in intracellular survival and virulence, which we wanted to understand.

Cryptococcal pathogenesis depends on a variety of virulence traits (33–35). One major virulence factor is capsule, but mitochondrial morphology does not play a role in the ability of C. neoformans to produce this structure. The ability of C. neoformans to cause disease also depends on its ability to survive at mammalian body temperature, a rare ability in fungi. While the *fzo1* mutant strain grows slightly slower at 37°C, this does not explain its complete inability to survive in the mouse lung environment. Another virulence factor is the formation of melanin. The *fzo1* mutant strain is deficient in melanin production at 37°C, but this is probably secondary to the slower growth at elevated temperature, as it melanizes fully at 30°C. Finally, as an intracellular pathogen, C. neoformans must be able to either prevent the respiratory burst or resist oxidative stress in phagocytes. The *fzo1* mutant strain is severely defective in growth in the presence of hydrogen peroxide, suggesting an explanation for its inability to survive within phagocytes and in mice. Further support for this model is our finding that the presence of ROS scavengers reverses the growth defect of the *fzo1* mutant within phagocytes. We suggest that increased mitochondrial fusion in WT C. neoformans results in an increased ability to clear ROS. This clearance, along with melanin production, mitigates the oxidative and nitrosative damage that cryptococcal cells experience within phagocytes. Together, these adaptations allow fungal cells to proliferate and cause disease *in vivo*.

Decreased mitochondrial tubularization causes a greater susceptibility of C. neoformans to oxidative and nitrosative stresses. We speculate that this is due to an inability of the mitochondrial network to undergo fusion, resulting in increased destruction of mitochondria with ROS-damaged mtDNA (10). This could directly impair well-characterized mitochondrial mechanisms for clearing ROS, such as manganese superoxide dismutase, cytochrome *c*, and reduced glutathione (36). Furthermore, decreased mitochondrial function could lead to an impaired production of NADPH, a key substrate for thioredoxin-, peroxiredoxin-, and glutathione-mediated ROS clearance (36, 37). We also speculated that strains with increased mitochondrial tubularization would correspondingly exhibit increased stress resistance, as has been observed *in vitro* for S. cerevisiae (38). Surprisingly, however, cells overexpressing *FZO1*, or lacking key mitochondrial fission proteins, resembled the WT in their ability to survive oxidative stress. This suggests mechanistic differences between the species, highlighting the danger of assuming that the basic biology of fungal pathogens will resemble that of model yeasts.

Although increased fusion did not improve resistance to individual stressors *in vitro*, we wondered whether it might still provide a survival advantage within host cells, either *in vitro* within macrophages or during mouse infections. However, mitochondrial fusion beyond WT levels (in the context of *FZO1* overexpression or defects in fission) did not promote cell survival in either of these settings. There are several possible explanations for this finding. One is that while increased tubularization is protective of cells, it is also detrimental to their viability or growth rate. This idea is supported by studies in S. cerevisiae, where enhanced mitochondrial fusion leads to defects in mtDNA segregation and cell division (1, 39, 40). It is also consistent with studies performed by the May laboratory, which suggested that C. gattii cell populations are protected by a subset of cells that demonstrate increased mitochondrial fusion, at the cost of their own ability to divide (19). Another explanation is that the KN99α parental strain, derived from a clinical isolate (41), already has optimal mitochondrial morphology for survival and proliferation in environments that impose oxidative stress; therefore, further increases in mitochondrial fusion do not assist in survival.

Mitochondria and mitochondrial processes have been considered for potential antifungal targets (3, 42), and mitochondria have been successfully targeted by antibiotics in other pathogens. Artemisinin, an antimalarial drug, causes a specific increase in ROS production in malarial and even some fungal mitochondria (43, 44). Another compound, atovaquone, kills malarial parasites by collapsing their mitochondrial membrane potential (45). Some mitochondrial pathways, such as those for β-oxidation and proline oxidation, have already been shown to be critical for cryptococcal virulence (46, 47). The mitochondrial respiratory chain (MRC) also plays a key role in drug resistance in C. neoformans, and the application of an MRC inhibitor enhances antifungal efficacy (48). Determining how the mitochondrial network maintains and controls its complex structure in C. neoformans will lay the foundation for targeting these processes.

We have defined the behavior and control of mitochondrial morphology in C. neoformans, a significant worldwide pathogen. These experiments are critical to understanding how mitochondria and their morphology play a role in cryptococcal virulence. Future studies to define the timeline of changes in cryptococcal mitochondrial morphology or the process of mitochondrial motility in single cells will provide insight on how C. neoformans adapts to the environment. Understanding these responses will be key to exploiting this crucial organelle for antifungal therapy.

## MATERIALS AND METHODS

### Gene identification, strain construction, and cell growth.

The cryptococcal gene encoding Fzo1 (CNAG_06688) was identified by BLASTp search of the C. neoformans genome using the S. cerevisiae Fzo1p protein sequence (YBR179C) (49). *DNM1*, *MDV1*, and *FIS1* (CNAG_01655, CNAG_01867, and CNAG_02519) were similarly identified by BLASTp searches, using the sequences of S. cerevisiae Dnm1p, Mdv1p, and Fis1p (YLL001W, YJL112W, and YIL065C, respectively). BLASTp searches of each cryptococcal gene yielded the original S. cerevisiae gene, showing that all are reciprocal homologs.

For strain construction, we used a split-marker strategy (50) with biolistic transformation of the C. neoformans KN99α strain (41) and confirmed all candidate transformants by PCR and the pattern of drug resistance (51). We first generated two independent *fzo1* deletion strains by replacing *FZO1* with a nourseothricin (NAT) resistance marker. To complement these mutants at the native site, we replaced the NAT coding sequence with the *FZO1* coding sequence, followed by its native terminator and a Geneticin (G418) resistance marker. We also generated two independent strains that overexpress *FZO1*. To do this, we replaced the native *FZO1* promoter with the cryptococcal *ACT1* promoter, in tandem with an upstream NAT marker to assist in screening. We also used the G418 marker to generate *dnm1*, *mdv1*, and *fis1* deletion strains, and we generated an *mdv1* deletion in KN99**a** cells. Finally, we generated a double-mutant strain by crossing the mating type **a** *mdv1* deletion strain to the Fzo1^OE^ strain on V8 mating medium and selecting *mdv1* × Fzo1^OE^ recombinants by their resistance to both G418 and NAT.

For all studies, C. neoformans strains were grown overnight in YPD medium (1% [wt/vol] Bacto yeast extract, 2% [wt/vol] dextrose, 2% [wt/vol] Bacto peptone in double-distilled water [ddH_2_O]) at 30°C with shaking at 230 rpm. For growth curves, cells were harvested at 1,000 × *g*, washed with phosphate-buffered saline (PBS), and adjusted to 1 × 10^5^ cells/ml for growth in YPD (30°C) or DMEM (37°C, 5% CO_2_).

### Mouse organ burden.

Overnight cultures of the desired strains were harvested by centrifugation (1,000 × *g*), washed with sterile PBS, and resuspended in PBS to 2.5 × 10^5^/ml. Groups of six 4- to 6 week-old female C57BL/6 mice (The Jackson Laboratory) were anesthetized by injection with 1.20 mg ketamine and 0.24 mg xylazine in 120 µl sterile water and intranasally inoculated with 1.25 × 10^4^ cryptococcal cells. Two mice were immediately sacrificed, and lung homogenates were prepared and plated on YPD agar (YPD medium, 2% [wt/vol] agar) plates to quantify the CFU. After 1 week, the remaining mice were sacrificed and similarly analyzed. Organ burden was analyzed by one-way analysis of variance (ANOVA) with Tukey’s *post hoc* test.

### Microscopy.

Strains were grown and washed in PBS as described above and diluted to a final density of 1 × 10^6^ cells/ml in 25 ml of prewarmed (37°C) DMEM (with or without 1 mM H_2_O_2_) in 75-ml tissue culture flasks. After incubation for 24 h at 37°C with 5% CO_2_, cells were again washed in PBS. To assay mitochondrial morphology, cells were then resuspended to 1 ml in PBS, stained for 1 h at 37°C with MitoTracker CMXRos (1 mM), and washed again with PBS. Images were collected with a Zeiss Axio Imager M2 fluorescence microscope with a Hamamatsu Flash4.0 CMOS camera, and at least 150 cells per sample were categorized in a blinded manner as having fragmented, tubular, or diffuse mitochondrial morphology. All experiments were performed with at least three independent biological replicates and analyzed using Fisher’s exact test of independence (52). Time course studies were performed the same way except that multiple tissue culture flasks were inoculated in parallel. At each time point, cells from one flask were sampled for CFU and then analyzed for morphology as described above. To assay capsule size, cells were resuspended at 1 × 10^6^ cells/ml in a 50% (vol/vol) solution of India ink. Images were collected with a Zeiss Axio Imager M2 fluorescence microscope with a Hamamatsu Flash4.0 CMOS camera.

### Dot plating.

Overnight cultures in YPD were harvested and washed in PBS as described above, adjusted to 10^7^ cells/ml, and serially diluted to final cell concentrations of 10^6^, 2.15 × 10^5^, 4.65 × 10^4^, and 10^4^ cells/ml. Four microliters of each dilution was spotted onto YPD and stress plates and grown at 30 and 37°C. To induce electron transport chain inhibition stress, YPD agar was supplemented with 75 µg/ml rotenone, 1 mM malonic acid, or 10 mM potassium cyanide (24). To assess growth using alternative carbon sources, YNB agar (0.67% [wt/vol] Yeast nitrogen base without amino acids, with ammonium sulfate, 2% [wt/vol] agar) was supplemented with 2% (wt/vol) glucose, maltose, sucrose, glycerol, ethanol, or sodium acetate (24). To induce cell wall stress, YPD agar was supplemented with calcofluor white at 0.050% (wt/vol). To impose oxidative and nitrosative stress, YNB agar with 2% (wt/vol) d-glucose and 25 mM sodium succinate was supplemented with 0.5 mM H_2_O_2_ or NaNO_2_. To assess melanin production, 5 μl of cells at 5.00 × 10^6^ cells/ml was spotted onto YNB agar plates supplemented with 1 mg/ml d-glucose, 1 mg/ml l glycine, 4 mg/ml KH_2_PO_4_, 0.46 mg/ml MgSO_4_·7H_2_O, 0.5 μg/ml d-biotin, 0.5 μg/ml thiamine, and 0.2 mg/ml l-3,4-dihydroxyphenylalanine (l-DOPA) (53). Melanization plates were incubated at 30 and 37°C for 2 and 3 days, respectively.

### XTT assay.

Overnight cultures in YPD were harvested and washed in PBS as described above, and one aliquot was heat killed (65°C, 5 min) as a control before dilution of all samples to 10^7^ cells/ml in prewarmed DMEM without phenol red (catalog no. 21063 029; Thermo Fisher). XTT [2,3-bis(2-methoxy-4-nitro-5-sulfophenyl)-2H-tetrazolium-5-carboxanilide] (catalog no. X6493; Thermo Fisher) and phenazine methosulfate (catalog no. P180858; Fisher) were added to the samples for final concentrations of 300 nM and 5 nM, respectively, followed by incubation for 3 h at 37°C and 5% CO_2_ with light vortexing every 30 min. Following incubation, cells were pelleted (1,000 × *g*), and the supernatant fraction was measured for absorbance at 450 nm (27, 54).

### Fungal intracellular survival.

THP-1 cells (1 ml of 3.50 × 10^4^ cells/ml in each well of 24-well plates) were differentiated in THP-1 medium (10% heat-inactivated fetal bovine serum, 48 µM 2-mercaptoethanol, 1 mM sodium pyruvate, 100 µM penicillin, 100 U/ml streptomycin in RPMI) with 25 nM phorbol myristate acetate (PMA) by incubating for 48 h at 37°C in 5% CO_2_, and then permitted to recover for 24 h in THP-1 medium without PMA (55). C. neoformans cells from overnight cultures in YPD were washed in PBS as described above, opsonized in 40% human serum in PBS (30 min at 37°C at 10^7^ cells/ml), washed in PBS, and resuspended in RPMI. Opsonized fungi were added to differentiated THP-1 cells at a multiplicity of infection (MOI) of 0.1 in three parallel plates, and the plates were incubated at 37°C and 5% CO_2_ for 1 h and washed with PBS; completion of the wash was considered 0 h of the survival study. One plate was immediately treated with water to lyse the macrophages and plated on YPD agar plates to quantify CFU. Two additional plates were incubated with THP-1 medium for lysis and analysis at 24 and 48 h, respectively. In some assays, ROS scavengers of 8 μg/ml bovine erythrocyte superoxide dismutase (catalog no. S5395; Sigma), 80 μg/ml bovine liver catalase (catalog no. 02100429; MP Biomedicals), and 100 mM d mannitol (catalog no. M1902; Sigma) were added to the THP-1 medium at 0 h and every 6 h after that during incubation (31). In all studies, fold changes in CFU were compared using one-way ANOVA with Tukey’s *post hoc* test.
