# Supplementary material for: Maintenance of Mitochondrial Morphology in Cryptococcus neoformans Is Critical for Stress Resistance and Virulence
Source: mBio. 2018 Nov 6;9(6):e01375-18. doi: 10.1128/mBio.01375-18 (PMC6222134; doi:10.1128/mBio.01375-18)
Supplement: FIG S1 [file mbo005184138sf1.pdf]

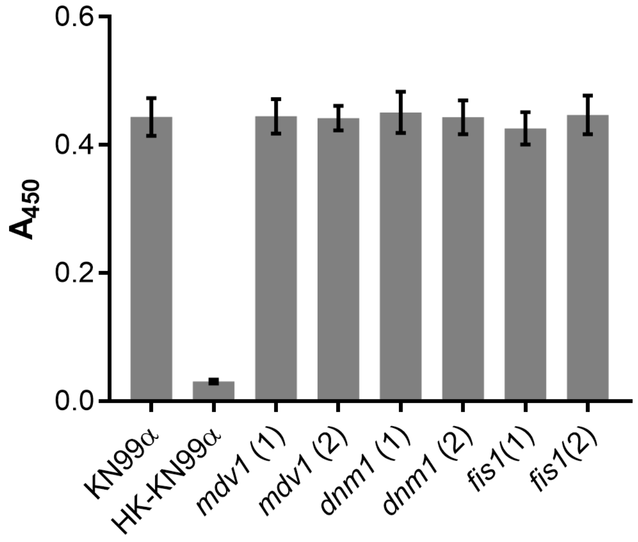

Figure S1: Metabolic activity of fungi. Plotted are the mean and SD of XTT assay results (A_{450}) for each indicated strain; HK denotes heat-killed cells. Results are shown for two independently derived stains for each fission mutant are shown and all results are representative of three biological replicate experiments.
