# Supplementary material for: Maintenance of Mitochondrial Morphology in Cryptococcus neoformans Is Critical for Stress Resistance and Virulence
Source: mBio. 2018 Nov 6;9(6):e01375-18. doi: 10.1128/mBio.01375-18 (PMC6222134; doi:10.1128/mBio.01375-18)
Supplement: FIG S2 [file mbo005184138sf2.pdf]

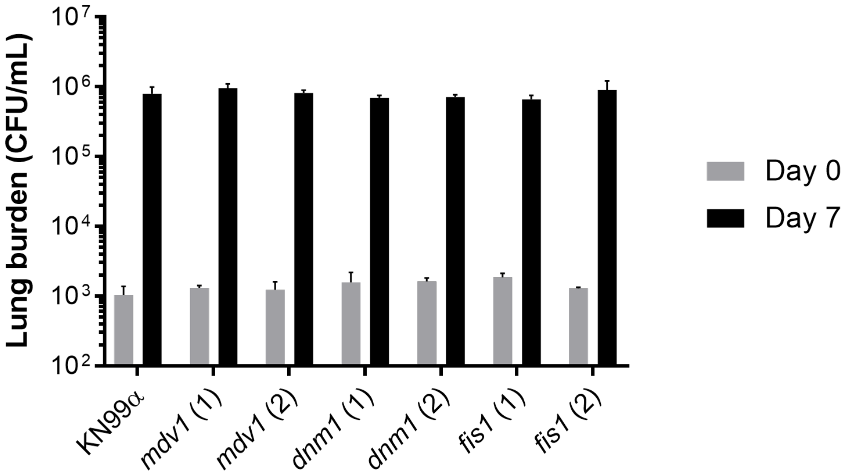

Figure S2: Mitochondrial fission mutants have no defect in virulence. Mouse lung burden in colony-forming units (CFU) after intranasal infection (see Methods) with the indicated fungal strains; two independent isolates of *mdv1*, *dnm1*, and *fis1* are shown. Gray, day 0 (n = 2); black, day 7 (n = 4). Mean and SEM are shown.
