# Supplementary material for: Maintenance of Mitochondrial Morphology in Cryptococcus neoformans Is Critical for Stress Resistance and Virulence
Source: mBio. 2018 Nov 6;9(6):e01375-18. doi: 10.1128/mBio.01375-18 (PMC6222134; doi:10.1128/mBio.01375-18)
Supplement: FIG S3 [file mbo005184138sf3.pdf]

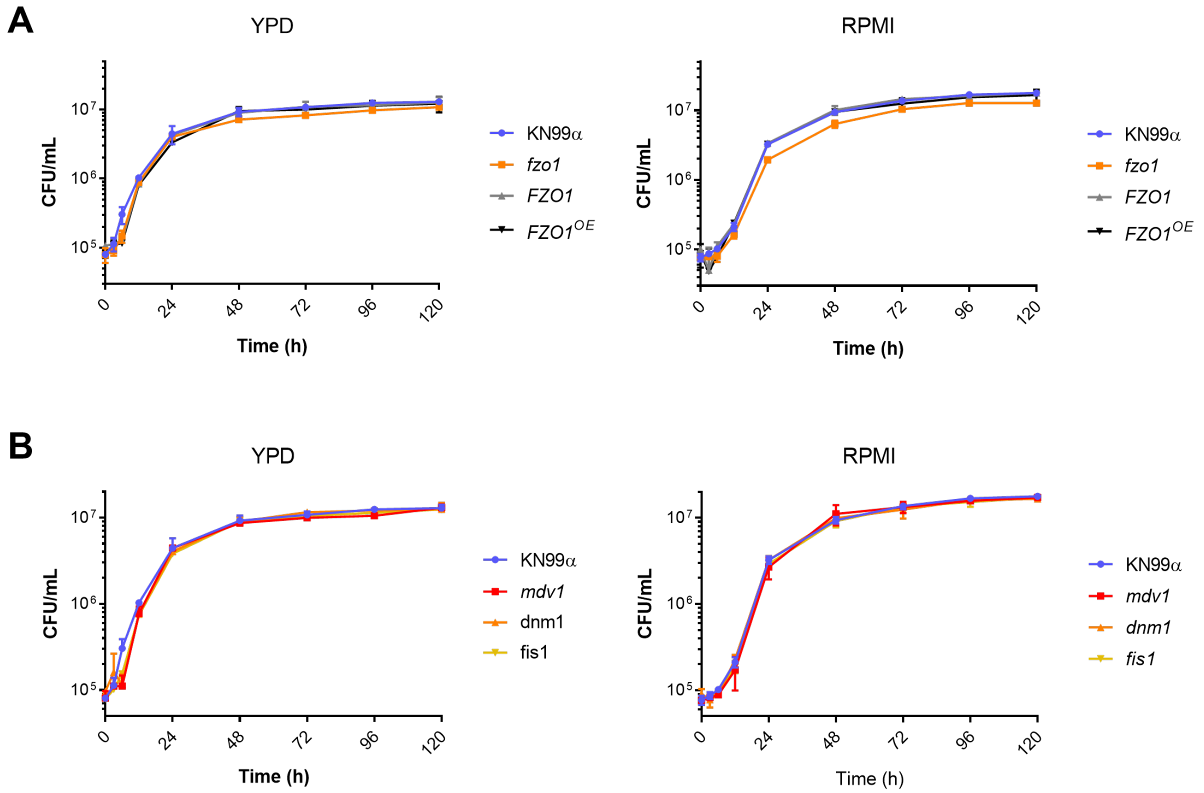

Figure S3: Growth curves in the indicated media of strains with altered *FZO1* expression (A) or deletions of genes involved in mitochondrial fission (B). YPD, YPD medium at 30 °C, room air; RPMI, RPMI medium at 37 °C, 5% CO_2. Mean and SD of triplicate time points are shown.
