# Supplementary material for: Maintenance of Mitochondrial Morphology in Cryptococcus neoformans Is Critical for Stress Resistance and Virulence
Source: mBio. 2018 Nov 6;9(6):e01375-18. doi: 10.1128/mBio.01375-18 (PMC6222134; doi:10.1128/mBio.01375-18)
Supplement: FIG S4 [file mbo005184138sf4.pdf]

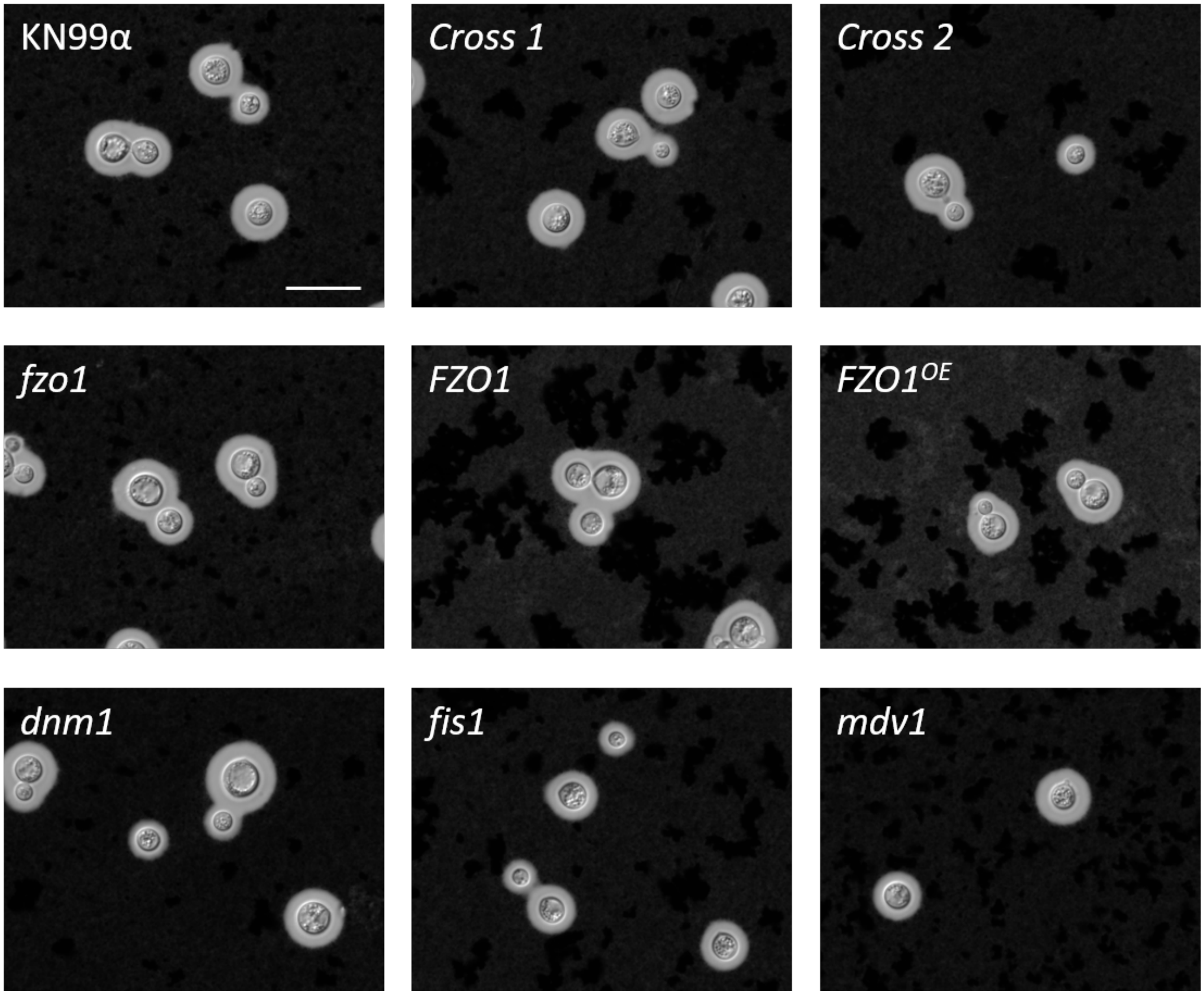

Figure S4: India ink stain of cells grown in capsule inducing conditions (DMEM, 37 °C, 5% CO_2) for 24 hours. Cross indicates *FZO1^{OE} mdv1* double mutants. Scale bar, 20 μm.
