# Supplementary material for: Maintenance of Mitochondrial Morphology in Cryptococcus neoformans Is Critical for Stress Resistance and Virulence
Source: mBio. 2018 Nov 6;9(6):e01375-18. doi: 10.1128/mBio.01375-18 (PMC6222134; doi:10.1128/mBio.01375-18)
Supplement: FIG S5 [file mbo005184138sf5.pdf]

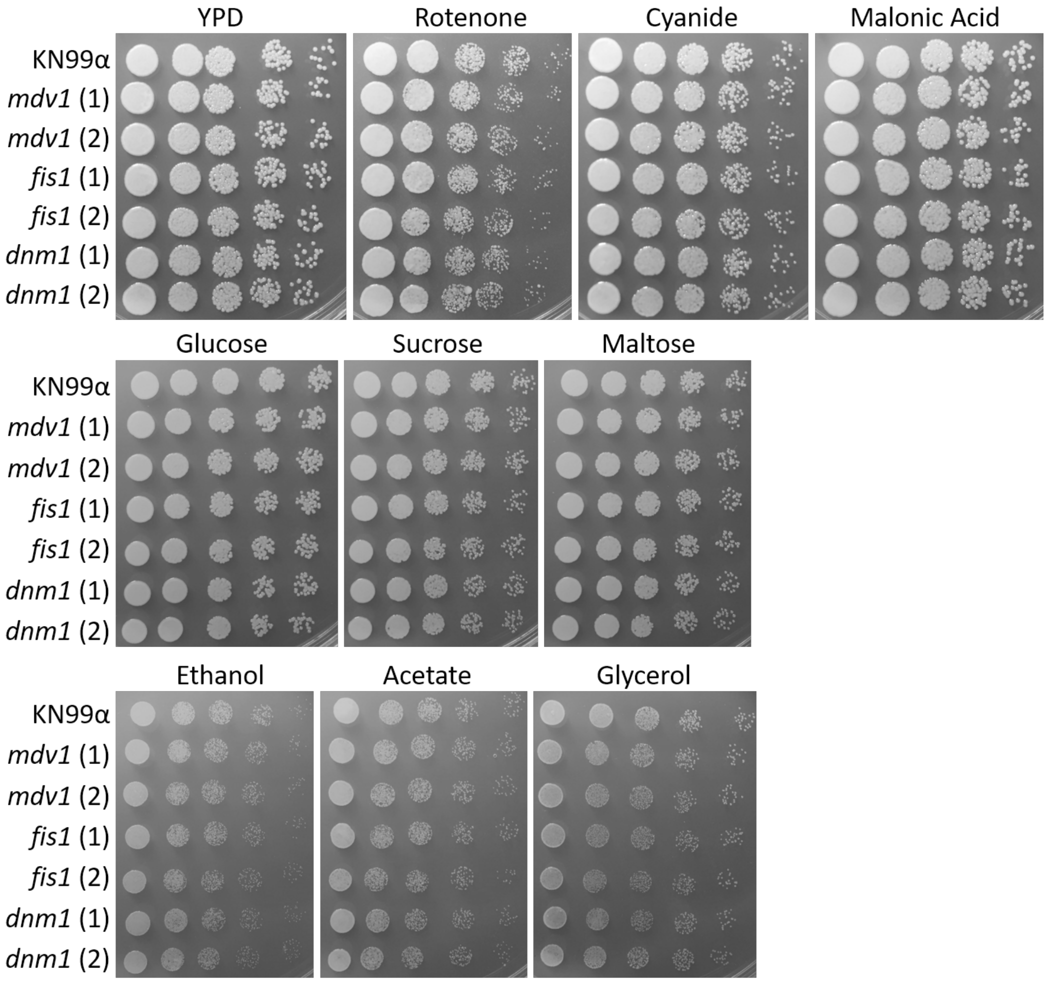

Figure S5: Stress phenotypes of mitochondrial morphology mutants on rich medium (YPD). Serial dilutions of the indicated strains were grown in the absence or presence of the indicated additives (see Methods for details).
