# Supplementary material for: Maintenance of Mitochondrial Morphology in Cryptococcus neoformans Is Critical for Stress Resistance and Virulence
Source: mBio. 2018 Nov 6;9(6):e01375-18. doi: 10.1128/mBio.01375-18 (PMC6222134; doi:10.1128/mBio.01375-18)
Supplement: FIG S6 [file mbo005184138sf6.pdf]

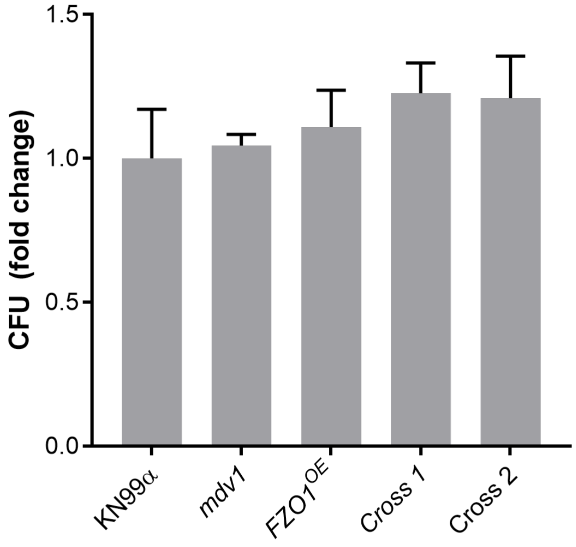

Figure S6: Survival of the indicated mutant strains that show increased tubularization after engulfment by macrophages. Cross 1 and 2 are independent *FZO1*^{OE} *mdv1* double mutants. For each strain, the ratio of CFU at 48 h to CFU at 0 h was normalized to this ratio for KN99 control cells (typically 1.5 to 2.0). Mean and SD are plotted, and results shown are representative of three biological replicate experiments.
